# Progress in Research on the Mechanisms and Therapeutic Targets of Ferroptosis in Peripheral Nerve Injury and Repair

**DOI:** 10.1080/17590914.2026.2678168

**Published:** 2026-05-27

**Authors:** Jian Ruan, Xin-Kun He, Song-Ou Zhang, Xiao-Feng Teng, Yiyang Hu, Hong Chen

**Affiliations:** a Department of Hand Surgery, Ningbo No.6 Hospital, Ningbo, Zhejiang, China; b Ningbo Clinical Research Center for Orthopedics, Sports Medicine & Rehabilitation, Ningbo, Zhejiang, China; c Department of Anesthesiology, Zhejiang Provincial People’s Hospital, Hangzhou, Zhejiang, China

**Keywords:** Dorsal root ganglion, ferroptosis, nerve regeneration, peripheral nerve injury, Schwann cells, therapeutic targets

## Abstract

Repair after peripheral nerve injury (PNI) faces major obstacles due to microenvironmental imbalance and neuronal loss. Ferroptosis, an iron-dependent cell death driven by lipid peroxidation, has emerged as a key pathological event in PNI, linking oxidative stress, mitochondrial dysfunction, and inflammation to regenerative failure. Targeting ferroptosis protects vital cells—such as Schwann cells and neurons—and ameliorates the regenerative niche, offering a promising therapeutic strategy. This review elucidates the mechanisms of ferroptosis in PNI, detailing its roles in Schwann cells, dorsal root ganglion neurons, and macrophages via core pathways including Nrf2/HO-1/GPX4 and ACSL4. We further evaluate current intervention strategies and their therapeutic efficacy. This synthesis provides novel insights into PNI pathology and guides the development of innovative treatments.

## Introduction: Clinical Challenges and Bottlenecks in Peripheral Nerve Injury Repair

Peripheral Nerve Injury (PNI) is a clinically common and disabling condition, frequently caused by trauma, compression, iatrogenic procedures, or metabolic disorders. It results in sensory loss, motor dysfunction, and intractable neuropathic pain in patients, severely impairing their quality of life and imposing a heavy socioeconomic burden (Jiang et al., 2026; Long et al., 2026; Rizvanović et al., 2026). Significant mechanistic disparities exist among neuropathies based on aetiology. For instance, diabetic neuropathy is characterized by chronic hyperglycemic insults leading to advanced glycation end-products accumulation, protein kinase C activation, and polyol pathway hyperactivity, resulting in persistent oxidative and endoplasmic reticulum stress (He et al., 2026; Jeong et al., 2026; Ye et al., 2024). This chronic pathological milieu starkly contrasts with acute traumatic PNI, which is primarily defined by immediate physical axon interruption and subsequent Wallerian degeneration. Ferroptosis, a novel form of regulated cell death named in 2012, is iron-dependent and driven by lipid peroxidation; morphologically, biochemically, and genetically, it is distinct from apoptosis, necrosis, and autophagy. In recent years, mounting evidence has indicated that ferroptosis is not merely a mode of cell death observed in vitro, but is also broadly implicated in the pathological processes of various neurological disorders. The pathogenic role of ferroptosis has been preliminarily substantiated in central nervous system disorders such as cerebral ischemia, cerebral hemorrhage, Alzheimer’s disease, and Parkinson’s disease (Du et al., 2025; Li & Jia, 2023; Yadav et al., 2023; Yao et al., 2026). Ferroptosis also plays a pivotal role within the peripheral nervous system. Emerging studies provide compelling clues in this regard, revealing the immense potential of ferroptosis as a novel therapeutic target for clinical intervention.

Preliminary studies have confirmed that in classic animal models of PNI—such as those involving the sciatic and facial nerves—characteristic biochemical alterations associated with ferroptosis are consistently observed at the injury site. These alterations include iron ion accumulation, glutathione depletion, GPX4 downregulation, and increased levels of lipid peroxidation products. Ferroptosis acts as a central nodal event linking various pathological processes following PNI—including oxidative stress, mitochondrial dysfunction, and inflammatory responses—to the ultimate outcomes of regenerative failure or chronic pain. This review aims to systematically organize and summarize the current progress in research regarding the role of ferroptosis in peripheral nerve injury and repair. We will begin by outlining the evidence implicating ferroptosis in PNI; subsequently, we will elucidate its specific functions and the complex molecular regulatory networks governing its activity within various cell types, such as Schwann cells and neurons. Finally, we will critically evaluate the diverse therapeutic strategies currently being developed to target key signaling nodes of ferroptosis, assessing their efficacy and potential for promoting nerve regeneration and alleviating neuropathic pain. This article aspires to offer novel perspectives for a deeper understanding of the pathological mechanisms underlying PNI, while simultaneously providing a theoretical foundation and conceptual framework for the development of innovative, anti-ferroptosis-based therapeutic strategies.

## Ferroptosis Contributes to Peripheral Nerve Injury

Direct evidence of ferroptosis’s involvement in peripheral nerve injury (PNI) initially emerged from biochemical and molecular analyses of classic injury models. As research progressed, investigators discovered that ferroptosis constitutes an early and critical pathological event following PNI. In classic animal models of PNI—such as sciatic nerve crush or transection, and facial nerve injury—researchers detected a series of characteristic biochemical alterations associated with ferroptosis within the injured nerve tissue, DRG, and even the spinal cord; temporally, these changes were closely correlated with the processes of Wallerian degeneration and nerve regeneration. The mechanism of local iron overload following PNI primarily stems from the rupture of microvessels at the injury site, leading to erythrocyte extravasation and hemolysis, which releases heme iron. Additionally, significant iron metabolism dysregulation occurs in Schwann cells and infiltrating macrophages during the phagocytosis of myelin debris and cellular remnants, resulting in substantial intracellular accumulation of Fe^2+^. The most direct evidence lies in the significant exacerbation of iron metabolic dysregulation and lipid peroxidation. Following injury, levels of Fe^2+^ and iron deposition increased markedly within the local injured nerve tissue and the corresponding DRG; concurrently, the levels of malondialdehyde (MDA) and 4-hydroxynonenal (4-HNE)—terminal products of lipid peroxidation—also rose in tandem (Liu et al., 2024; Zhou et al., 2026). Excess Fe^2+^ catalyzes the generation of ROS through the Fenton reaction, directly attacking polyunsaturated fatty acids on the cell membrane and triggering a lethal chain reaction of lipid peroxidation. This indicates that the injury triggered an iron-dependent Fenton reaction, thereby precipitating lethal lipid peroxidation. Correspondingly, the cellular antioxidant defense system was severely compromised. Crucial glutathione (GSH) levels were significantly depleted post-injury, while the protein and mRNA levels of glutathione peroxidase 4 (GPX4)—a core enzyme responsible for executing antioxidant functions—exhibited significant downregulation in both the injured sciatic nerves and DRG neurons (Gao et al., 2022; Guo et al., 2021; Liu et al., 2024). The loss of GPX4 activity rendered the accumulated lipid peroxides incapable of being timely detoxified, ultimately leading to the disintegration of cellular membranes (Wang et al., 2025). These alterations in biochemical markers are not isolated phenomena; rather, they are intimately linked to neurological functional deficits and morphological damage. In models of facial nerve injury, the emergence of the aforementioned ferroptosis markers was accompanied by a concomitant loss of motor function in the facial muscles. Intervention via intrathecal or local administration of ferroptosis-specific inhibitors not only effectively reverses these biochemical abnormalities but also significantly promotes functional recovery of the facial or sciatic nerves, while improving axonal regeneration and myelin repair (Gao et al., 2022; Guo et al., 2021). The causal relationship between the inhibition of ferroptosis and the promotion of functional recovery provides the most compelling in vivo validation of the pathogenic role of ferroptosis in PNI. To elucidate the specific cellular targets and mechanism of action of ferroptosis in PNI, in vitro studies focusing on Schwann cells and DRG neurons have offered a more refined mechanistic perspective. These two cell types constitute the core units of peripheral nerve regeneration, and their ultimate fate directly determines the success or failure of the regenerative process (Bosch-Queralt et al., 2023; Nocera & Jacob, 2020; Renthal et al., 2020). In Schwann cells, treatment with classical ferroptosis inducers—specifically Erastin (which inhibits System Xc^-^, thereby disrupting GSH synthesis) or RSL3 (which directly inhibits GPX4 activity)—successfully induces cell death, accompanied by morphological alterations and biochemical signatures characteristic of ferroptosis. This form of cell death can be specifically rescued by the ferroptosis inhibitors Ferrostatin-1 or deferoxamine, but not by inhibitors of apoptosis or necrosis (Gao et al., 2022). Studies have demonstrated that the overexpression of c-Jun can enhance the resistance of Schwann cells to Erastin-induced ferroptosis—potentially through mechanisms involving the upregulation of Nrf2—thereby offering novel strategies for preserving Schwann cell viability by modulating endogenous protective pathways (Gao et al., 2022). Furthermore, in the context of diabetic neuropathy, a high-glucose environment can also induce ferroptosis in Schwann cells, accompanied by mitochondrial dysfunction; notably, pharmacological agents such as honokiol have been shown to suppress this process by activating the AMPK/SIRT1/PGC-1α signaling axis (Hu et al., 2023).

DRG neurons are similarly susceptible to ferroptosis. Acrylamide exposure can induce ferroptosis in DRG neurons—manifested by GPX4 downregulation, GSH depletion, and lipid peroxidation—leading to reduced neuronal viability and impaired neurite outgrowth; notably, pretreatment with Ferrostatin-1 can significantly reverse these detrimental effects (An et al., 2022). In models of hypoxia, hypoxia-inducible factor-1α (HIF-1α) protects DRG neurons against ferroptosis and promotes their survival by upregulating the expression of SLC7A11 and GPX4 (An et al., 2024). Furthermore, Schwann cell-derived exosomes can inhibit neuronal ferroptosis by delivering the MFG-E8 protein to modulate the intracellular PPARγ/p53/SAT1/ALOX15 signaling pathway, thereby highlighting the critical role of intercellular communication in combating ferroptosis (Cao et al., 2025). These cell-level investigations not only confirm that Schwann cells and neurons constitute key targets of ferroptosis but also elucidate the common downstream endpoints of ferroptosis activation—as well as their upstream regulatory networks—under various pathological stimuli, including mechanical injury, metabolic toxicity, and hypoxia. Fibroblasts and infiltrating neutrophils are increasingly recognized for their importance in PNI pathogenesis (He et al., 2022; Ren et al., 2024; Yon et al., 2023), yet their involvement in ferroptosis remains poorly understood. Fibroblasts are pivotal in forming the fibrotic scar tissue that physically impedes axonal regeneration, while infiltrating neutrophils orchestrate the initial inflammatory response and clear myelin debris (Dahlin, 2023). Although current studies have predominantly focused on Schwann cells and neurons, the potential for ferroptosis to occur in fibroblasts and neutrophils—and its subsequent impact on the extracellular matrix remodeling or inflammatory resolution—represents a critical gap in the field. Future studies should extend beyond traditional targets to investigate whether modulating ferroptosis in these stromal and immune cells could reshape the regenerative microenvironment, thereby offering novel combinatorial therapeutic strategies.

**Figure 1.**
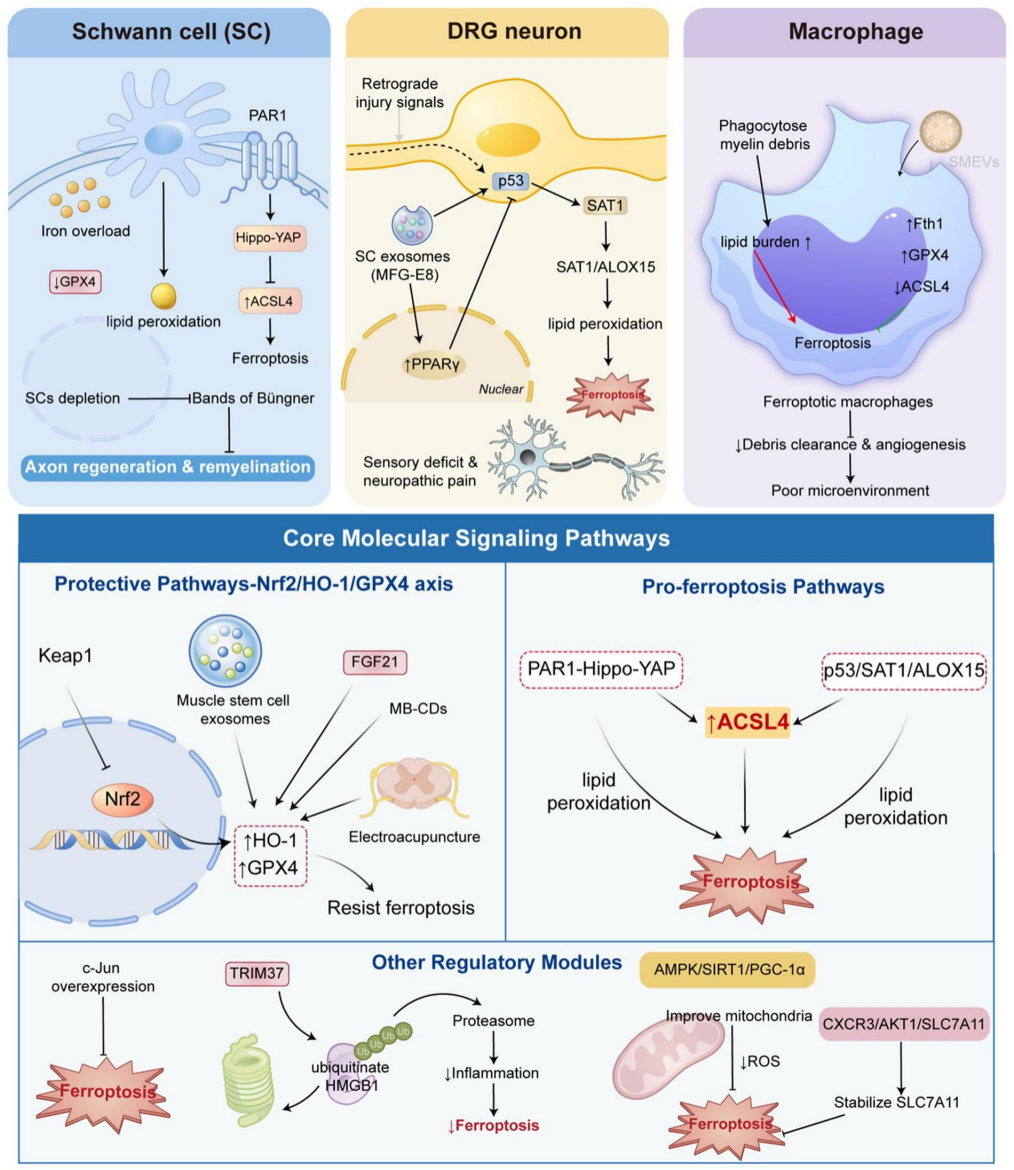
The core mechanisms of ferroptosis in peripheral nerve injury.

Beyond hypothesis-driven research targeting specific molecules and pathways, omics analyses have provided systematic evidence regarding the role of ferroptosis in PNI (Aiki et al., 2018; Gu et al., 2022). By performing transcriptome sequencing on neural tissues at various time points following PNI and integrating these data with known ferroptosis-related gene sets through bioinformatics analysis, researchers have successfully identified core hub genes involved in the ferroptosis process subsequent to PNI. One such study, utilizing Weighted Gene Co-expression Network Analysis and machine learning algorithms to screen gene expression data following sciatic nerve injury in mice, successfully identified six ferroptosis-related hub genes closely associated with the progression of PNI: Cdkn1a (p21), Cdh1 (E-cadherin), Hif1a, Hmox1, Nfe2l2 (Nrf2), and Tgfb1 (Zhang et al., 2023). This list of genes holds profound biological significance. Notably, Nrf2 and its downstream target Hmox1 constitute the core of the canonical pathway for combating oxidative stress and ferroptosis (O’Rourke et al., 2024; Peng et al., 2025; Zhang, 2025; Zhou et al., 2024). Hif1a is upregulated during hypoxic stress and has been demonstrated to confer resistance to ferroptosis by activating SLC7A11 (An et al., 2024). Cdkn1a participates in cell cycle regulation and stress responses; Tgfb1 acts as a pivotal regulator of fibrosis and inflammation; and Cdh1 is implicated in cell adhesion and the maintenance of the Schwann cell phenotype (Chen et al., 2023; Girardi et al., 2022; Manousakis et al., 2023; Ryan et al., 2024). The identification of these genes provides independent validation—at the transcriptome level—that ferroptosis-related pathways are significantly activated following PNI; furthermore, it organically links ferroptosis to other critical biological processes occurring post-PNI, such as oxidative stress, hypoxic adaptation, inflammatory responses, cell cycle arrest, and the formation of a fibrotic microenvironment. This suggests that ferroptosis is not an isolated event, but rather a central node embedded within the complex pathological network of PNI, regulating multiple interconnected processes of injury and repair (Figure 1).

## The Core Mechanisms of Ferroptosis in Peripheral Nerve Injury

The destructive role of ferroptosis in PNI is mediated through its actions on the injured region and adjacent key functional cells, operating via a complex network of molecular signaling pathways (Bosch-Queralt et al., 2023; Krishnan et al., 2024; Wu et al., 2025). Elucidating these cellular targets and regulatory pathways is fundamental to understanding the pathological contributions of ferroptosis and to developing precision therapies.

### Key Cellular Targets

Schwann cells (SCs) are the glial cells of the peripheral nervous system and play an indispensable role following nerve injury: they undergo dedifferentiation and proliferation, clear myelin debris, and align to form the Bands of Büngner—structures that guide axonal regeneration—while simultaneously secreting neurotrophic factors; thus, they serve as the architects of the nerve regeneration microenvironment (Fuentes-Flores et al., 2023; Sun et al., 2023; Yao et al., 2024). However, SCs are exceptionally susceptible to ferroptosis. Studies have demonstrated that following sciatic nerve crush injury or facial nerve injury, SCs exhibit significant iron overload, downregulation of GPX4 expression, and lipid peroxidation. Ferroptosis in SCs—whether artificially induced or triggered by pathological conditions—leads to their massive depletion, thereby preventing the formation of effective regenerative tracks (Gao et al., 2022, 2025; Hu et al., 2023). Activation of Protease-Activated Receptor 1 (PAR1) can upregulate the expression of ACSL4 via the Hippo-YAP signaling axis, thereby specifically promoting ferroptosis in SCs and significantly inhibiting myelin regeneration (Wu et al., 2025). Consequently, protecting SCs from ferroptosis is a critical prerequisite for maintaining their supportive functions and ensuring effective axonal regeneration and successful remyelination.

DRG neurons serve as the primary afferent neurons for sensory input; the survival of their cell bodies and the regenerative capacity of their axons directly determine whether sensory function can be restored (Donovan et al., 2025; Feng et al., 2023; Navia-Pelaez et al., 2023). DRG neurons also represent a key cellular target for ferroptosis. Following PNI, retrograde injury signals can trigger the activation of ferroptosis pathways within DRG neurons, leading to irreversible neuronal death and functional loss—a process that underlies permanent sensory deficits (An et al., 2022; Liu et al., 2024). Beyond directly causing neuronal depletion, ferroptosis in DRG neurons has also been established as a critical mechanism in the pathogenesis of neuropathic pain. The release of ferroptosis-induced lipid peroxidation products and inflammatory mediators can aberrantly activate and sensitize pain pathways. For instance, Schwann cell-derived exosomes can upregulate neuronal PPARγ by delivering the MFG-E8 protein, thereby inhibiting the pro-ferroptosis p53/SAT1/ALOX15 pathway and consequently alleviating pain (Cao et al., 2025). Therefore, inhibiting ferroptosis in DRG neurons serves a dual purpose: protecting the neuronal pool to support regeneration and alleviating intractable neuropathic pain.

During the Wallerian degeneration phase following PNI, macrophages recruited to the injury site are responsible for the efficient clearance of degenerating myelin and axonal debris, thereby clearing obstacles to regeneration (Oshima et al., 2023; Sun et al., 2023, 2024). Simultaneously, through M1/M2 phenotypic switching and the secretion of cytokines, these macrophages modulate the inflammatory microenvironment and promote angiogenesis. Recent studies have revealed that macrophages that have phagocytosed myelin debris experience a drastic increase in their lipid metabolic burden, rendering them more susceptible to ferroptosis (Huo et al., 2025). Research involving *Salvia miltiorrhiza*-derived extracellular vesicles (SMEVs) has demonstrated that these ferroptotic macrophages suffer from functional impairment, exhibiting diminished clearance capabilities and a reduced capacity to promote angiogenesis. However, by upregulating Ferritin Heavy Chain 1 (Fth1) and GPX4 within macrophages while simultaneously downregulating ACSL4, SMEVs can effectively inhibit ferroptosis, thereby preserving the macrophages’ scavenger function and fostering angiogenesis conducive to nerve regeneration (Huo et al., 2025). This indicates that macrophage ferroptosis is an indirect yet significant factor influencing the quality of the neuroregeneration microenvironment.

### Core Molecular Signaling Pathways

The fate of ferroptosis is determined by the balance between intracellular pro-survival and pro-death signaling networks. In the context of PNI, the roles of several core pathways are particularly prominent. Nrf2 serves as a central regulator of the cellular antioxidant response. Under conditions of oxidative stress, Nrf2 translocates to the nucleus, where it activates the expression of a series of cytoprotective genes, including heme oxygenase-1 (HO-1) and GPX4 (Liu et al., 2024; Tosyalı et al., 2023). This pathway has been demonstrated to be a common therapeutic target for various neuroprotective strategies aimed at combating ferroptosis. Muscle stem cell-derived exosomes protect Schwann cells by activating the Nrf2/HO-1/GPX4 axis through the inhibition of Keap1 (a negative regulator of Nrf2) (Liu et al., 2024); FGF21 suppresses mitochondrial damage-induced ferroptosis via the ERK/Nrf2/GPX4 pathway (Yan et al., 2024), and mung bean-derived carbon dots (MB-CDs) also protect Schwann cells through this same pathway (Zheng et al., 2025). Similarly, the alleviation of neuropathic pain by electroacupuncture relies on the activation of the Nrf2 pathway within the spinal cord (Xue et al., 2024). Consequently, the Nrf2/HO-1/GPX4 axis represents a core endogenous defense mechanism for enhancing the resistance of neural cells to ferroptosis.

Acyl-CoA synthetase long-chain family member 4 (ACSL4) is responsible for esterifying polyunsaturated fatty acids and incorporating them into membrane phospholipids, thereby providing substrates for lipid peroxidation; it acts as a critical “accelerator” determining cellular susceptibility to ferroptosis (Sun et al., 2026; Wu et al., 2025). In the context of PNI, at least two distinct pathways have been confirmed to upregulate ACSL4, thereby driving ferroptosis. First, the thrombin receptor PAR1 is activated following injury; acting via the Hippo-YAP signaling pathway, this activation leads to reduced nuclear translocation of YAP, thereby lifting its transcriptional repression of ACSL4. Consequently, ACSL4 is upregulated, promoting ferroptosis in Schwann cells (Wu et al., 2025). Second, within DRG neurons, DNA damage or cellular stress can activate p53, which in turn upregulates SAT1 and ALOX15, thereby promoting arachidonic acid metabolism and lipid peroxidation; this p53/SAT1/ALOX15 pathway is intimately linked to the execution of ferroptosis (Cao et al., 2025). Inhibiting these pathways can effectively reduce ACSL4 levels or activity, thereby curbing ferroptosis.

Beyond the aforementioned core pathways, a complex regulatory network participates in the fine-tuning of ferroptosis following PNI. The transcription factor c-Jun, a pivotal regulator of Schwann cell dedifferentiation and regeneration, confers enhanced resistance to ferroptosis in Schwann cells upon its overexpression (Gao et al., 2022). Conversely, the E3 ubiquitin ligase TRIM37 inhibits the release of the damage-associated molecular pattern (DAMP) protein HMGB1 by promoting its ubiquitination-mediated degradation, thereby attenuating inflammation and suppressing ferroptosis in Schwann cells (Zhou et al., 2026). In the context of diabetic neuropathy, activation of the AMPK/SIRT1/PGC-1α axis contributes to improved mitochondrial function and reduced ROS production, thereby inhibiting ferroptosis in Schwann cells (Hu et al., 2023). Furthermore, the CXCR3/AKT1/SLC7A11 pathway—identified in models of intracerebral hemorrhage—suggests that AKT signaling may influence ferroptosis by regulating the stability of SLC7A11; the role of this pathway in PNI warrants further investigation (Hu et al., 2025).

### Upstream Triggers and Interactions

The occurrence of ferroptosis is not an isolated event, but rather the collective outcome—and an amplifier—of a series of pathophysiological changes following PNI.

Mitochondrial dysfunction constitutes a critical upstream event triggering ferroptosis. Mitochondria serve as the primary site for ROS generation and act as a pivotal node in iron metabolism. Following PNI, the ensuing energy crisis and calcium overload can lead to the collapse of mitochondrial membrane potential, morphological abnormalities, and functional impairment. Compromised mitochondria not only generate copious amounts of ROS—thereby promoting lipid peroxidation—but may also release factors involved in ferroptosis signaling. This release can trigger the loss or inactivation of anti-ferroptosis proteins, such as FSP1, thereby tightly coupling mitochondrial damage with the execution of ferroptosis (Yan et al., 2024).

Oxidative stress and inflammation, in turn, form a vicious cycle with ferroptosis. PNI directly generates substantial amounts of ROS, triggering oxidative stress that attacks cellular lipid membranes. Concurrently, injury-activated inflammatory cells (specifically M1-type macrophages) release a plethora of inflammatory cytokines—such as TNF-α and IL-1β—which can further suppress the activity of System Xc- or promote iron accumulation. Conversely, lipid peroxidation products (4-HNE) and damage-associated molecular patterns (HMGB1) released by ferroptotic cells act as potent activators of inflammation and inducers of oxidative stress, thereby continuously amplifying the injury signals (Gao et al., 2025; Hu et al., 2023; Zheng et al., 2025). Ferroptosis-driven oxidative stress establishes a feed-forward loop via Transient Receptor Potential Ankyrin 1 (TRPA1) to sustain chronic pain. Oxidative stress upregulates M-CSF in Schwann cells, expanding resident macrophages and amplifying TRPA1 sensitization. Activated Schwann cell TRPA1 then perpetuates this cycle, linking ferroptotic damage directly to pain hypersensitivity (De Logu et al., 2021).

Hypoxia plays a complex role within the PNI microenvironment. Local circulatory disturbances resulting from the injury inevitably lead to hypoxic conditions. Interestingly, under conditions of moderate hypoxia, the hypoxia-inducible factor HIF-1α becomes stabilized; it subsequently transcriptionally upregulates genes such as SLC7A11 and GPX4, serving as an adaptive cytoprotective response to counteract ferroptosis (An et al., 2024). This reveals that, under pathological conditions, cells attempt to balance the detrimental effects of hypoxia against the risk of ferroptosis by activating HIF-1α; however, persistent and severe hypoxia will ultimately overwhelm this protective mechanism.

In summary, ferroptosis plays a profound role in the regenerative failure and pain pathogenesis observed following PNI. It achieves this by precisely targeting key functional cell types—including Schwann cells, neurons, and macrophages—and by disrupting the equilibrium of core signaling pathways, such as the Nrf2/GPX4 and ACSL4 pathways. This process is driven and modulated by multiple upstream factors—including mitochondrial dysfunction, oxidative stress, inflammation, and hypoxia—constituting a critical link within the complex pathological network of PNI.

## Therapeutic Strategies and Potential Targets for Ferroptosis

Given the central role of ferroptosis in the pathology of PNI, targeting key steps in the ferroptosis pathway has garnered significant attention from researchers. Current research efforts primarily encompass the development of small-molecule inhibitors, cell-derived therapeutics, novel biomaterials, and natural compounds, as well as the exploration of physical and genetic intervention strategies.

Specific small-molecule inhibitors of ferroptosis serve as primary tools for validating its pathological role and exploring its therapeutic potential. Among these, Ferrostatin-1 (Fer-1) and Liproxstatin-1 (Lip-1)—classic antioxidant-type ferroptosis inhibitors—have demonstrated clear protective effects across various models of PNI and neuropathic pain (Liu et al., 2020; Tong et al., 2023; Xiao et al., 2021). Through their potent free-radical scavenging activity, Fer-1 and Lip-1 directly neutralize lipid peroxidation radicals and interrupt the chain reactions of lipid peroxidation, thereby exerting their effects during the downstream execution phase of ferroptosis (Bao et al., 2022). In an acrylamide-induced model of DRG neuronal injury, pretreatment with Fer-1 effectively reversed the acrylamide-triggered downregulation of GPX4, depletion of GSH, and accumulation of lipid ROS; this significantly enhanced neuronal viability and preserved the growth capacity of neuronal processes, thereby demonstrating the direct protective effect of ferroptosis inhibition on neurons (An et al., 2022). In models of neuropathic pain induced by chronic constrictive injury (CCI) of the sciatic nerve or spinal nerve ligation (SNL), intrathecal administration of Lip-1 significantly attenuated mechanical and thermal hyperalgesia in rats. The underlying mechanism involves the suppression of injury-induced ferroptosis-specific changes within the spinal cord and DRGs—such as reduced GPX4 expression and increased lipid peroxidation (Guo et al., 2021). Similarly, in a model of inflammatory pain induced by complete Freund’s adjuvant (CFA), intrathecal administration of Lip-1 likewise exerted an analgesic effect by inhibiting ferroptosis within the spinal cord and DRGs (Deng et al., 2023). These studies not only confirmed the involvement of ferroptosis in various forms of neuronal injury and pain but also demonstrated the feasibility of using broad-spectrum ferroptosis inhibitors as a proof-of-concept therapy, thereby laying the groundwork for the subsequent development of inhibitors with greater tissue specificity and stability.

Iron chelators serve as a complement to antioxidant inhibitors. Since ferroptosis is initiated by Fe^2+^-driven Fenton reactions, directly chelating free iron represents a logical and potent interventional strategy. Deferoxamine, a canonical iron chelator, has been investigated in PNI models. Administration of DFO effectively reduces iron deposition at the injury site, thereby suppressing lipid peroxidation and preventing the subsequent inactivation of the GPX4 antioxidant system (Zhang et al., 2023). Unlike Fer-1 or Lip-1, which primarily act downstream to scavenge free radicals, iron chelators target the upstream by eliminating the catalytic substrate essential for ferroptosis propagation. Developing targeted delivery systems for iron chelators to specifically mitigate localized iron surges following PNI remains a highly promising direction for future research.

Extracellular vesicles—particularly exosomes—serve as natural carriers for intercellular communication, capable of delivering bioactive substances such as proteins, lipids, and nucleic acids; they are characterized by low immunogenicity, excellent biocompatibility, and inherent targeting capabilities (Cunha et al., 2024; Kim et al., 2024; Wang et al., 2024). Exosomes derived from mesenchymal stem cells have garnered significant attention in the field of neural repair. Studies have demonstrated that exosomes derived from muscle-derived stem cells (MDSCs) can effectively repair sciatic nerve injuries (Xu et al., 2020; Xun et al., 2021). Mechanistically, upon endocytosis by Schwann cells, these exosomes deliver specific signaling molecules that inhibit the Keap1 protein, thereby stabilizing and activating the pivotal antioxidant transcription factor, Nrf2. The activated Nrf2 subsequently translocates to the nucleus, where it upregulates the expression of a cascade of antioxidant and anti-ferroptosis genes—including HO-1 and GPX4—ultimately bolstering Schwann cells’ resistance to ferroptosis and promoting neural regeneration (Liu et al., 2024). This highlights the systemic regulatory advantage of stem cell-derived exosomes, which achieve multi-target protection by modulating endogenous defense pathways. Exosomes secreted by Schwann cells themselves also play a pivotal role in modulating the neural microenvironment (Sun et al., 2023; Zhu et al., 2023). Studies have revealed that Schwann cell-derived exosomes are enriched with the Milk Fat Globule-EGF Factor 8 (MFG-E8) protein. Following neuronal injury, these exosomes can be taken up by adjacent dorsal root ganglion (DRG) neurons. The MFG-E8 delivered via these exosomes is capable of upregulating the expression of Peroxisome Proliferator-Activated Receptor gamma (PPARγ) within the neurons. The activation of PPARγ subsequently inhibits p53—a key upstream regulator of pro-ferroptotic pathways—and downregulates its downstream effector molecules, SAT1 and ALOX15. This action reduces the peroxidation of arachidonic acid, thereby protecting neurons from ferroptosis. This intercellular rescue signaling effectively alleviates neuropathic pain (Cao et al., 2025). Plant-derived extracellular vesicles (EVs) have emerged as a promising area of research due to their abundant sources and low production costs (Dad et al., 2021; Kim et al., 2022). For instance, extracellular vesicles extracted from the traditional Chinese medicine Danshen (SMEVs) have been demonstrated to effectively promote peripheral nerve regeneration. A unique feature of SMEVs is their primary action on macrophages located at the site of injury. By upregulating Ferritin Heavy Chain 1 (Fth1—responsible for iron storage and reducing the toxicity of free iron) and GPX4 within macrophages, while simultaneously downregulating the pro-ferroptotic factor ACSL4, SMEVs specifically inhibit macrophage ferroptosis. Consequently, surviving macrophages are able to maintain their highly efficient phagocytic function and pro-angiogenic capabilities, thereby creating a clean, well-vascularized microenvironment conducive to nerve regeneration (Huo et al., 2025). This exemplifies an ingenious strategy for indirectly promoting regeneration by modulating the fate of immune cells.

Novel biomaterials offer a dual functionality for nerve repair, providing both physical support and biochemical regulation. Mung bean-derived carbon dots (MB-CDs) represent a class of nanomaterials characterized by their diminutive size and excellent biocompatibility. Studies have shown that MB-CDs serve not only as scaffold materials for nerve regeneration but also possess significant intrinsic biological activity. They are efficiently internalized by Schwann cells, where they activate the intracellular Nrf2 signaling pathway. This activation subsequently upregulates the expression of HO-1 and GPX4, thereby bolstering the antioxidant and anti-ferroptotic defenses of Schwann cells and promoting their survival and functional performance within the hostile environment of an injury site (Zheng et al., 2025). This opens up new avenues for the development of “smart” biomaterials that integrate both nerve-guiding capabilities and neuroprotective activities.

Active ingredients extracted from traditional Chinese medicines or natural plant sources constitute a rich reservoir for the discovery of novel ferroptosis inhibitors. Honokiol is a natural biphenyl compound extracted from Magnolia officinalis that has demonstrated protective effects in models of diabetic peripheral neuropathy. Its mechanism of action involves the activation of the AMPK/SIRT1/PGC-1α signaling axis within Schwann cells. On one hand, the activation of this pathway enhances mitochondrial biogenesis and function, thereby improving energy metabolism and reducing mitochondrially derived ROS; on the other hand, it directly bolsters cellular antioxidant capacity. Consequently, it comprehensively inhibits ferroptosis and functional impairment in Schwann cells under high-glucose conditions (Hu et al., 2023). Such natural products typically possess characteristics such as multi-target activity, mild action, and minimal side effects, offering promising prospects for clinical translation.

**Figure 2.**
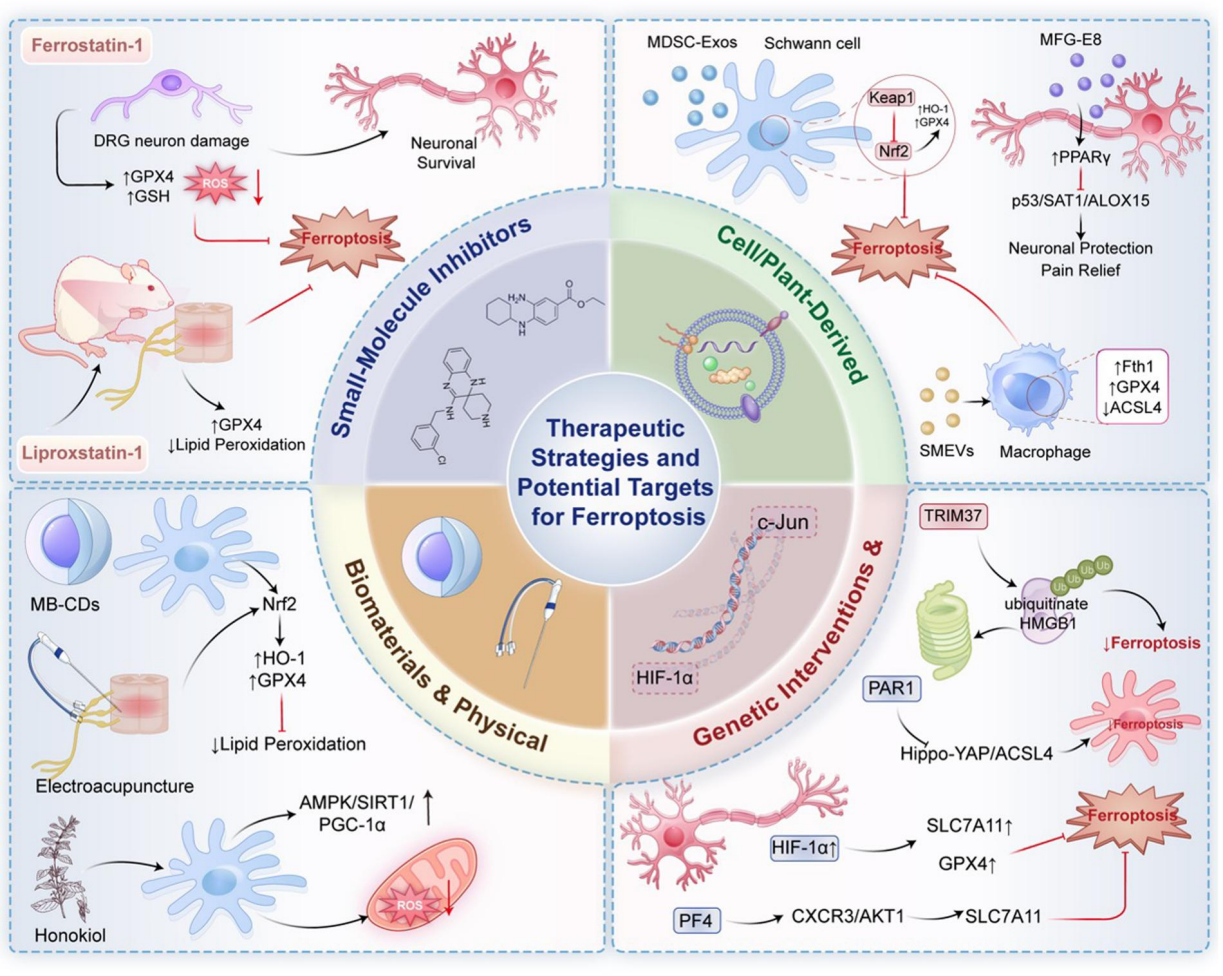
Therapeutic strategies and potential targets for ferroptosis.

As a non-invasive physical therapy modality, electroacupuncture is widely utilized in pain management (Chen et al., 2024; Tian et al., 2024). Studies have demonstrated that electroacupuncture treatment can significantly alleviate chronic neuropathic pain, and its analgesic molecular mechanisms are closely linked to the inhibition of ferroptosis in spinal cord neurons. Electroacupuncture stimulation activates the Nrf2 signaling pathway within pain-associated spinal cord segments, upregulating the expression of downstream proteins—such as GPX4—thereby bolstering neuronal antioxidant defenses and suppressing the occurrence of lipid peroxidation and ferroptosis (Xue et al., 2024). This provides a modern molecular biological explanation for traditional physical therapies and suggests that the activation of endogenous anti-ferroptosis pathways constitutes a key mechanism underlying the analgesic effects of electroacupuncture. In Schwann cells, the overexpression of the transcription factor c-Jun enhances cellular resistance to Erastin-induced ferroptosis and promotes facial nerve regeneration (Gao et al., 2022). Furthermore, the overexpression of the E3 ubiquitin ligase TRIM37 inhibits inflammation and ferroptosis—thereby alleviating nerve injury—by promoting the ubiquitination-mediated degradation of the injury-associated molecule HMGB1 and consequently reducing its release (Zhou et al., 2026). In dorsal root ganglion neurons, the overexpression of HIF-1α via viral vectors mimics the protective effects of hypoxia, upregulating SLC7A11 and GPX4 to counteract ferroptosis (An et al., 2024). In models of cerebral hemorrhage, platelet factor 4 (PF4) has been shown to inhibit ferroptosis by stabilizing SLC7A11 through the activation of the CXCR3/AKT1 pathway; a similar strategy holds potential for exploration within the context of PNI (Hu et al., 2025). Finally, the knockdown of protease-activated receptor 1 (PAR1) using siRNA blocks the activation of its downstream Hippo-YAP/ACSL4 signaling pathway, thereby attenuating ferroptosis in Schwann cells and promoting myelin regeneration (Wu et al., 2025). In a Schwann cell model infected with the HHV-7 virus, the knockdown of Cox4i2—a protein associated with mitochondrial complex IV—was shown to reduce ROS production via the ERK pathway, thereby inhibiting virus-triggered ferroptosis and apoptosis (Gao et al., 2025). Although these gene-level manipulations are currently utilized primarily for mechanistic investigations, advancements in gene delivery technologies hold the promise of enabling the development of precise gene therapy strategies in the future. While therapeutic strategies targeting ferroptosis have demonstrated encouraging prospects in preclinical studies, the field currently faces several critical challenges and limitations that urgently require resolution through future research. The efficacy of most strategies has been validated solely within models of standardized injury during the acute or subacute phases; their restorative effects in cases of chronic nerve injury or injuries with complex etiologies remain unclear. Furthermore, existing interventions lack sufficient spatiotemporal precision. There is a severe dearth of comparative studies and investigations into the synergistic effects of different strategies. Whether the combined application of strategies involving distinct mechanisms yields additive or synergistic benefits—and how to determine the optimal therapeutic regimen—remain unexplored areas (Figure 2).

## Summary and Outlook

In summary, ferroptosis—defined as an iron-dependent form of regulated cell death driven by lipid peroxidation—has been established as a pivotal pathological event following PNI. Far from existing in isolation, it is deeply integrated with a multitude of interconnected pathological processes—including oxidative stress, mitochondrial dysfunction, and neuroinflammation—which collectively led to the loss or dysfunction of key functional cell types, such as Schwann cells, neurons, and macrophages. Consequently, ferroptosis serves as a central nexus linking the initial injury to adverse outcomes such as failed regeneration and chronic neuropathic pain. Existing studies—spanning from animal models and cellular mechanisms to signaling pathways—have begun to delineate the complex regulatory network governing ferroptosis in the context of PNI, wherein imbalances within pathways such as Nrf2/GPX4 and ACSL4 play decisive roles. Therefore, the targeted inhibition of ferroptosis—whether through pharmacological, biomaterial-based, or genetic approaches—and particularly the achievement of precise regulation across different cell types, has emerged as a highly promising new strategy for promoting nerve regeneration and functional recovery.

Despite these promising prospects, the field continues to face numerous challenges. Foremost among them is the need for further elucidation regarding spatiotemporal specificity. The dynamic changes and mutual interactions of ferroptosis across different post-injury time windows and within various cell types remain incompletely understood; this knowledge gap limits the development of spatiotemporally regulated therapeutic strategies. Furthermore, the path toward clinical translation is arduous and protracted. The vast majority of promising interventions remain in the preclinical research phase; their long-term in vivo safety, optimal routes of administration, and efficacy in large animal models urgently require systematic evaluation. Moreover, disease-specific mechanisms warrant deeper investigation. In PNI cases stemming from diverse etiologies, the upstream signaling pathways triggering ferroptosis—as well as the specific cell types susceptible to it—may vary; consequently, future efforts must focus on developing targeted, individualized therapeutic strategies. Finally, multimodal combination therapy represents an inevitable trend. Anti-ferroptosis therapy alone may be insufficient to achieve complete nerve regeneration. Future research should explore integrating such therapies with existing pro-regenerative modalities—such as neurotrophic factor delivery, conductive or biomimetic scaffold materials, and cell transplantation—to formulate synergistic treatment regimens. By doing so, we can simultaneously safeguard cell survival while more actively guiding axon regeneration, myelination, and functional reconstruction, ultimately providing more effective solutions for the clinical management of PNI.
